# Out of hospital Cardio-pulmonary arrest - Is there a role for the primary healthcare teams?

**DOI:** 10.1186/s13584-017-0161-4

**Published:** 2017-06-28

**Authors:** Shlomo Vinker

**Affiliations:** 1 Department of Family Medicine, Sackler Faculty of Medicine, Tel Aviv University, Tel Aviv, Israel; 2 Medical Director Office, Leumit Health Services, Tel Aviv, Israel

**Keywords:** Cardiopulmonary resuscitation, Delivery of Health Care, Quality of Health Care, Primary Health Care, Cardiopulmonary Arrest

## Abstract

Out of hospital cardiac arrest (OHCA) remains a major cause of morbidity and mortality. The survival rates are poor and even more frustrating are the rates of neurologically favorable outcomes at hospital discharge. In a recent IJHPR article, Einav et al. concluded that many primary care clinics are underequipped and the physicians underprepared to initiate life-saving services.

The chance of having an OHCA in a primary care clinic is very low. But although the impact is small, primary care teams as well as other out-of-hospital healthcare personal should be familiar with the telephone number for summoning emergency medical services (EMS), be aware of the location of the defibrillator in their clinic, and know how to use it.

The literature about effective ways to keep long-standing competencies in cardiopulmonary resuscitation among medical personnel outside the hospital is scarce. It is very difficult to evaluate the actual effectiveness of interventions on better outcome; the events are rare and unique in their nature and it hard to generalize the conclusions.

The “chain of survival” concept involves a series of steps that should be taken at the scene in the community: early recognition of symptoms and activation of an emergency response system; early bystander cardiopulmonary resuscitation; rapid defibrillation, if needed; early advanced cardiac life support and integrated post-resuscitation care. In this “chain” there is an important role for healthcare personal in the community via improving their own skills and performance and via a deeper involvement in the education of the public.

We should take all the needed steps so that community clinic personnel can be role models for effective and successful out of hospital cardiac resuscitation (OHCR).

## Background

Despite decades of research and medical training and education, cardiac arrest remains a major cause of morbidity and mortality. The survival rates are poor and even more frustrating are the rates of neurologically favorable outcomes at hospital discharge. In a recent IJHPR article, Einav et al. reported the findings of a small survey that was administered to primary care physicians working in community clinics in Israel and viewed as preliminary by the authors [1]. The questionnaire used was generated by the authors and the validation process was minimal. The survey had been distributed among 2400 primary care physicians via several mailing lists and web-based forums yielding a response rate of only 7.7% (185/2400). They concluded that many primary care clinics are underequipped and the physicians staffing them underprepared to initiate life-saving services, and that steps must be taken to rectify this situation.

Although it would be desirable at some point to have a study that explores the subject more extensively than is possible in a pilot study such as this, the topic of out of hospital cardiopulmonary resuscitation (OHCR) given in healthcare community facilities is worthy of discussion. The organizational, as well as, educational dilemma of: “How can we maintain the competencies of our clinic teams so that they will have a high level of preparedness for rare but life threatening situations?” has been raised and should be discussed.

## Out of hospital cardiopulmonary resuscitation by healthcare staff

Most of the epidemiological studies about OHCR divide the scene of arrest between private residences and public locations and hence it is very difficult to extract data about the number of OHCRs actually done in community clinics. For example, Gaieski et al. found that in the years 2008–2012 in Philadelphia the majority (76.2%) of OHCR cases occurred in a residence, while only 7.5% occurred in a healthcare facility [2]. In a multivariate analysis, being resuscitated in healthcare facility did not give superior outcomes, but it seems that the majority of OHCRs were in nursing homes and not in community clinics. Fan et al. from Hong Kong also noted that nearly 30% of OHCRs occurred in a home for the aged or nursing home for the elderly and with poorer outcomes [3]. Even Nishiyama et al. cited by Einav et al. are speaking about the possibility of early prodromal symptoms that may cause patients to seek help from their primary physician but fail to show the rate of “in clinic” OHCRs or to demonstrate any advantage of in-clinic resuscitation [4]. In a large and comprehensive United States survey only 2% (620/31,689) of persons who experienced OHCR had been resuscitated in the physician office/clinic [5]. The authors cite a relatively high (27%) survival rate in comparison to OHCR in other arenas, but surely the overall impact of this on overall OHCR outcomes is very low.

So in conclusion we can say that the chance of having an OHCR in a primary care clinic is very low. Furthermore, from the public health perspective, the impact of the OHCR in primary care clinics on the overall survival and outcome of OHCR is low and this is one of the reasons that automated, simple to activate, defibrillators have been distributed in public places other than healthcare facilities. But although the impact is small, primary care teams as well as other out-of-hospital healthcare personal should be familiar with the telephone number for summoning EMS, be aware of the location of the defibrillator in their clinic, and know how to use it in order to perform an effective cardio-pulmonary resuscitation (CPR).

## Maintaining competencies of cardiopulmonary resuscitation in community healthcare staff

Keeping knowledge, competencies and performance of the medical staff is a generalized and huge problem, especially in the current era of endless and ongoing generation of new scientific data. Being able to react and perform an effective cardiopulmonary resuscitation is even more complicated to maintain, as it is not only the individual doctor who needs to be familiar with the theoretical background and operational skills but also team-work training as well as adequate and ready-to-use equipment.

In a survey conducted among all primary care health centers in Finland (51% response rate), only a minority considered resuscitation training in their health center to be sufficient and systematic; and in health centers with an appointed person in charge of resuscitation training, the training was more often regular [6]. In Denmark direct mail to family physicians improved knowledge of changes in basic life support guidelines and thus might help to facilitate the translation of this knowledge into clinical practice [7].

The literature about effective ways to keep long-standing competencies in cardiopulmonary resuscitation among medical personnel outside the hospital is scarce. This could be due to several reasons: training should involve the clinic staff as a team, and teams are dynamic; it is very difficult to evaluate the actual effectiveness of interventions on better outcome; the events are rare and unique in their nature and it hard to generalize the conclusions. For these reasons we should look for new ways of continuous, cost effective training of individuals and teams in cardiopulmonary resuscitation.

## Summary

The “chain of survival” concept involves a series of steps that should be taken at the scene in the community: early recognition of symptoms and activation of an emergency response system; early bystander cardiopulmonary resuscitation; rapid defibrillation, if needed; early advanced cardiac life support and integrated post-resuscitation care. In this “chain” there is an important role for healthcare personal in the community via improving their own skills and performance and via a deeper involvement in the education of the public.

Various successful public health strategies have been implemented in different healthcare systems [8]. Specifically, the implementation of publicly available automatic external defibrillators (AEDs) and training of public bystanders to both deliver CPR and use AEDs. In some countries emergency non-medical first responders (e.g. police, firefighters) have been equipped and trained to use AEDs. The use of cellular phone applications can alert the public to find the nearest CPR trained individual. Various strategies have been shown to improve survival, but differing degrees of implementation have led to a disparity in survival rates.

## Conclusion

We should take all the needed steps so that community clinic personnel can be role models for effective and successful OHCR.

## Abbreviations

AED: Automatic external defibrillator
CPR: Cardiopulmonary resuscitation
EMS: Emergency medical services
OHCA: Out-of-hospital Cardiac Arrest
OHCR: Out-of-hospital Cardiac Resuscitation
